# Removal of Total Petroleum Hydrocarbons from Contaminated Soil through Microwave Irradiation

**DOI:** 10.3390/ijerph17165952

**Published:** 2020-08-17

**Authors:** Kanghee Cho, Eunji Myung, Hyunsoo Kim, Oyunbileg Purev, Cheonyoung Park, Nagchoul Choi

**Affiliations:** 1 Research Institute of Agriculture and Life Sciences, Seoul National University, Seoul 08826, Korea; kanghee1226@hanmail.net; 2 Department of Energy and Resource Engineering, Chosun University, Gwangju 61452, Korea; ej6865@naver.com (E.M.); star8538@naver.com (H.K.); oyunbileg@chosun.kr (O.P.); cybpark@chosun.ac.kr (C.P.)

**Keywords:** microwave, total petroleum hydrocarbons, vapor stripping, particle size, power density

## Abstract

In this study, we investigated the removal mechanism of total petroleum hydrocarbons (TPH) from soil by microwave heating. TPH contaminated soil was investigated to determine the desorption behavior of five carbon number-based fractions of TPH. The applied operating microwave power density influenced the final temperature that was reached during heating. For low operating power density applications, microwave effectiveness was limited due to the soil’s dielectric properties, which exhibited a direct relationship with temperature variation. Soil particle distribution could be attributed to permeability, which significantly influenced the evaporation of contaminated soil during the microwave treatment. The results indicate that the activation energy was correlated with the influence of particle size. The removal efficiency of the coarse soil reached 91.1% at 15 min, whereas that of fine soil was low. A total of 30 min had passed, and a removal efficiency of 71.2% was found for the fine soil. Residual TPH concentration was decreased when irradiation time was increased with a removal rate dependent on soil temperature variation. The surface functional groups of the contaminated soil were influenced by microwave irradiation, and changes in the hydrocarbon fraction affected contaminant removal.

## 1. Introduction

Petroleum hydrocarbons (hydrocarbons that result from petroleum products such as oil, gasoline, or diesel fuel) pollution has become a global matter of environmental concern [1]. Petroleum is released into the environment as a result of industrial discharge, storage tank leaks, and other accidents [2]. There is substantial petroleum discharge into soil and aquifers. Petroleum contaminated soil is considered to be hazardous waste that causes local and diffuse pollution in the environment [3]. Total petroleum hydrocarbons (TPH) are composed of a complex mixture of saturated hydrocarbons (primarily paraffins, 60–80%) and aromatic hydrocarbons (20–40%) [4,5].

Most TPH contaminants are biodegradable over time, but bioremediation has poor bioavailability and a long degradation period because of the inefficient air permeability of TPH- contaminated soil and mass transfer efficiency [6]. In addition, the high TPH removal efficiency observed for thermal remediation is hardly achievable in such a short time by using bioremediation [7]. Thermal remediation is a common technology for treating TPH-contaminated soils. Thermal desorption (from 100 to 600 °C) is remediation technology that utilizes an external heat source to enhance the evaporation of volatile organic contaminants so that they can be removed from the soil into the gas phase. On the other hand, incineration (up to 1000 °C) techniques were proposed for the remediation of TPH-contaminated soil, particularly to reduce remediation time and complete removal capability [8,9,10]. Incineration can decompose TPH and may remove contaminants from TPH-contaminated soil [11]. However, the operational cost, energy consumption, and structural deterioration of soil have restricted its application [12].

Microwaves have been recognized as cost-effective and time-saving and include the homogeneous heating of contaminant substances [13]. Microwave heating is widely applied for the treatment of hazardous waste such as petroleum-contaminated soils [14], and it has attracted great attention in the environmental field [15]. The microwave heating principle is based on the transformation of microwave energy adsorbed by the irradiated substances into heat [16]. The microwave absorption capability of dielectric substances is based on the dielectric properties of the substances, which in turn depend on the dielectric constant and dielectric loss factor [17]. Dielectric properties are critical factors for remediation efficiency. Microwave heating remediation, relative to thermal remediation technology such as incineration and thermal desorption, significantly minimizes operation cost requirements [18]. In practical use, the cost-effectiveness of remedial treatment is a very important concern. Microwave heating remediation of soils contaminated with hydrocarbons under different heating conditions (soil texture and moisture) and operating conditions (supplied power and time) was investigated by Falciglia et al. (2017), who assessed specific energy consumption and energy costs from modelling. Specifically, the comparison of calculated costs with those of other remediation technologies for hydrocarbon contaminated soils showed that the obtained short remediation times and energy costs make microwave heating a deliverable alternative to conventional thermal desorption or physical-chemical techniques [19].

Most investigations focused on the efficiency of TPH removal, and only a few investigated the influence of the dielectric properties of soils [20,21]. The application of different soil textures (e.g., sand, silt, and clay) significantly influences microwave remediation; consequently, the specific surface area influences the interaction of compounds with the soil and the remediation of hydrocarbon pollutants [22]. In addition, during microwave heating, soil moisture is gradually converted into water vapor, thereby resulting in a contaminant desorption phenomenon by distillation, which contributes to contaminant removal [23,24]. The effects of vaporization on the process of TPH-contaminated soil remediation are of great importance. This means that permeability in soil plays a major role in contaminant vaporization. Because soil permeability significantly influences soil interaction during desorption, on the basis of the above considerations, soil particles were divided into two different particle sizes (coarse soil, >0.106 mm; fine soil, <0.106 mm). In this study, the microwave treatment of soils artificially contaminated by diesel fuel was examined using a laboratory-scale microwave system; the aim was to confirm the remediation mechanism of TPH in soil by vapor stripping and to investigate the behavior of different TPH fractions.

## 2. Materials and Methods

### 2.1. Soil Characterization

**Figure A1:**
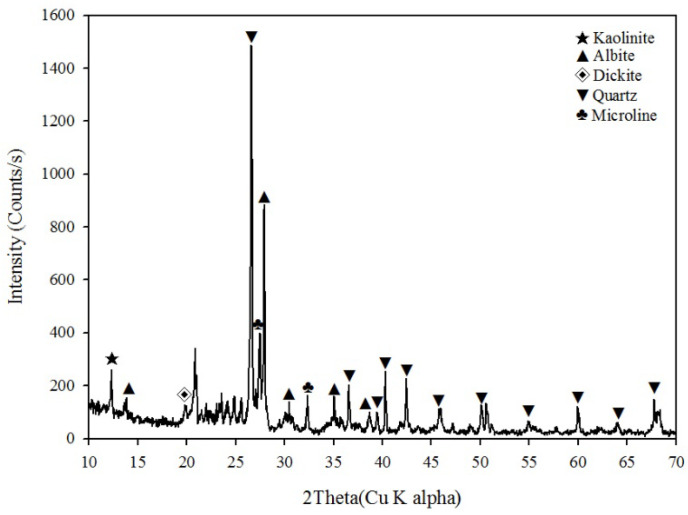
XRD pattern of soil sample.

The removal efficiencies of contaminants are affected by soil particle size. The soil surface area significantly influences contaminant soil interactions in adsorption and desorption processes. Considering that the moisture content of clay is higher than that of sand, sandy soil was selected for the experiments. Soil particle size analysis showed that it consisted of sand (79.80%), silt (16.50%), and clay (3.65%), which represented the textural classification of sandy loam soil. Mineral composition analysis of the contaminated soil using XRD (X-ray diffraction) revealed that it consisted of kaolinite, albite, dickite, quartz, and microcline (Figure A1). Quartz was dominant in the soil, which is hydrophobic. The major components of the soil were Si, Al, Fe, and K; small amounts of Ca and Mg were also detected (Table 1). The primary physical properties of the soil were a pH of 6.9, organic matter content of 1.63%, and moisture content of 4.57% (Table 2).

### 2.2. Contamination Procedure

The selected soil samples were artificially contaminated with diesel fuel. Commercially available diesel fuel (GS Caltex, Korea) was used for the artificial contamination procedure. The soil was dried, homogenized, and sieved to remove large particles. The diesel contamination procedure was performed by mixing a pollutant solution of diesel fuel with soil (at a mass ratio of 1:9). The mixtures were stored in a fume hood for one week. Thermal gravimetric analysis (TGA) results for the contaminated soil revealed a weight loss of 7.8% under a nitrogen environment. The contaminated soil revealed a TPH concentration of 988.4 mg/kg for bulk soil. Soil particles were divided into two size fractions (with different concentrations of TPH), coarse soil (>0.106 mm, 1222.1 mg/kg) and fine soil (<0.106 mm, 2222.9 mg/kg).

### 2.3. Microwave Experiments Conditions and Procedures

**Figure 1:**
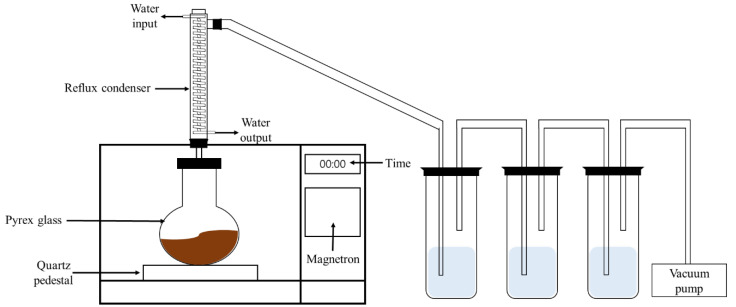
Schematic of laboratory-scale microwave apparatus.

Microwave heating was conducted using a system that was constructed for the treatment of hydrocarbon-containing solids, a schematic of which is shown in Figure 1. The top of the microwave was connected to a gas collection system, which consisted of three condenser traps and a vacuum pump. Vapor was collected from the condenser. Microwave irradiation was performed using a laboratory-scale apparatus, prepared by modifying a 2.45 GHz domestic microwave oven. The flask was placed in the center of the microwave system and irradiated. The inner temperature of the sample was measured using a type-K thermocouple that was axially inserted up to the middle of the soil sample. The surface temperature of the sample was also measured by using the type-K thermocouple. After the experiment, contents were cooled to ambient temperature. Sample weight was measured after microwave irradiation.

Enhanced microwave heating treatment was investigated for microwave power density. For each experiment, the contaminated soil sample was treated at a power of 800 W for different irradiation times of up to 30 min. The temperature profiles were investigated for microwave power density (0.5, 0.9, and 1.3 kW/kg) and particle size characteristics (±0.106 mm). Contaminant removal efficiency for particle size and bulk soil was investigated by microwave irradiation time. Each experiment was performed in duplicate, and the mean values of residual contaminant concentrations as a function of treatment time were obtained for each soil.

(1) $$R=\frac{\left(C_{0}-C\right)}{C_{0}}\times 100$$

where C_0_ is the initial contaminant concentration in soil (mg/kg), and C is the residual contaminant concentration in soil after microwave treatment (mg/kg).

### 2.4. Analysis Method

#### 2.4.1. Contaminated Soil Characterization Analysis

The pH of the soil was analyzed by mixing it with deionized water at a ratio of 1:5 (soil:deionized water). TGA Q500 (TA instrument, New Castle, DE, USA) was performed at a heating rate of 10 °C/min under N, and the scan range was from approximately 900 °C. The sample was subjected to XRD analysis with X’Pert Pro MRD (Panalytical, Amsterdam, The Netherlands). Cu Kα radiation was used at an acceleration voltage of 40 kV and a current of 30 mA. The 2θ section from 10° to 70° was analyzed for the soil. The elemental composition of the contaminated soil was determined with X-ray fluorescence (XRF) spectrometry RIX 2000 (Rigaku, Tokyo, Japan). The surface chemistries of the contaminated and treated soil samples were studied using Fourier-transform infrared spectroscopy Nicolet 6700 (Thermo Fisher Scientific, Waltham, MA, USA).

#### 2.4.2. Extraction and Analytical Methods

TPH concentration was measured with a gas chromatograph equipped with a flame ionization detector (GC-FID). The same procedure was used for all samples before and after microwave irradiation. First, 20 g of soil was mixed with 20 g of anhydrous Na_2_SO_4_ in a 200 mL borosilicate glass beaker, and 100 mL of dichloromethane was added. The mixture was sonicated in an ultrasonic apparatus Sonics Vibra-Cell (SONICS, Newtown, CT, USA) for 6 min and then filtered through a Buchner funnel using 5B filter paper. The final residue was collected in a 2 mL vial as the TPH sample. The TPH sample was injected by an auto-sample injector into the Agilent 7890B GC (Agilent, Santa Clara, CA, USA) equipped with a capillary column HP-Ultra 2 (Agilent, Santa Clara, CA, USA). TPH concentration in the samples was quantified against a calibration curve prepared using a standard TPH solution (AccuStandard, New haven, CT, USA). TPH determination in each sample was performed in duplicate with a relative error of less than 3%. In order to better understand the effect of the microwave heating treatments on the removal mechanisms, TPH (C10–C40) were divided into five fractions according to carbon numbers on the basis of the TPH Criteria Working Group (TPHCWG) method, as follows: disel range organics C10–C16 (DRO1), C16–C22 (DRO2), C22–C28 (DRO3), and oil range organics C28–C34 (ORO1), C34–C40 (ORO2). Diesel concentration was initially adsorbed on soil before the heating treatment was measured as TPH fractions (C10–C40), and a related percentage was calculated as the ratio between the concentration of a single TPH fraction and the total concentration of C10–C40.

## 3. Results

### 3.1. Temperature Profiles by Microwave Power Density

**Figure 2:**
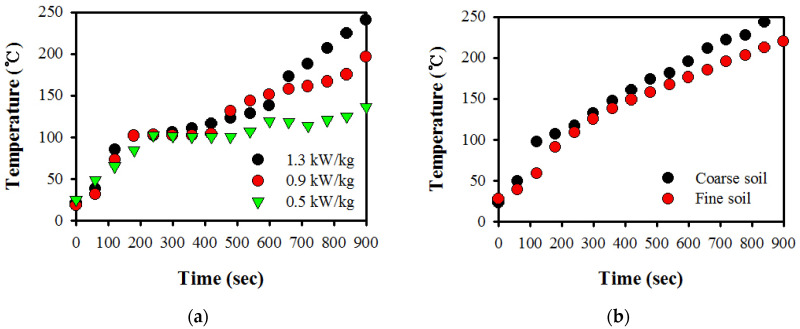
Temperature profiles of soil for (**a**) microwave power density and (**b**) different particle sizes with time during microwave irradiation.

The temperature profiles of the soil over time during microwave treatment at the investigated microwave power density series of 0.5, 0.7, 0.9, and 1.3 kW/kg for contaminated bulk soil are shown in Figure 2a. The microwave power density used in the operation had considerable influence on the remediation of soil contaminated with TPH [13,23]. For all the conditions, temperature increased with increasing power density. A maximal temperature of approximately 240.7 °C was reached at 1.3 kW/kg irradiation, whereas a maximal increase of approximately 136.4 °C was reached at 0.5 kW/kg. Temperature rapidly increased at the beginning of irradiation, and was stabilized after 200 s, which corresponded with approximately 100 °C. This stabilized temperature behavior, which depended on temperature, was kept almost constant until the evaporation of moisture content in the soil occurred because of the evaporation effect generated by the heat transfer between soil matrix and water [24]. A large increase in temperature was observed after 400 s because of the ability of the soils to convert the absorbed microwave energy into heat with a progressive increase in heat absorption [25]. In addition, the temperature profiles of the two different particle sizes (±0.106 mm) were measured over time by using 1.3 kW/kg, as shown in Figure 2b. Difference in temperature was observed in a temperature range of approximately 30 °C. At different temperatures, the increase in temperature over time was inversely proportional to the specific heat capacity (C_p_) and density (ρ) of the soil. The absorbed microwave power was converted to heat, and heating rate (∆T/∆t) was given by the following equation [26]:

(2) $$\frac{∆T}{∆t}=\frac{P}{\left(C_{p}\cdot \rho \right)}$$

where P is adsorbed power per unit volume (W/m^3^), C_p_ is the heat capacity of the medium (kJ/kg/°C), and ρ is the density of the medium (kg/m^3^).

Fine sand could be due to low permeability, which significantly influenced the evaporation of the soil sample during the thermal treatment, thereby resulting in a limitation of the heat flow. For coarse sand, heat flow spread more to the various layers of the soil, which resulted in a higher heating rate. Falciglia et al. (2011) found that soil texture influences the temperature profiles in soil by using a tubular electric furnace [21]. The results show that the variation in the temperature rise curve with coarse sand (500–840 µm) and clay (<4 µm) was different and in the order of clay > coarse sand with a difference of up to 20 °C. However, Falciglia et al. (2015) reported that when the temperature profiles of soil over time during microwave irradiation were investigated for medium sand (200–350 µm), fine sand (75–200 µm), silt (10–75 µm), and clay (<4 µm), the temperature of the four investigated soils followed the order of medium sand ≈ fine sand > silt ≈ clay between two groups of soils with a maximal difference of up to 65 °C [22]. Conventional thermal processing of heat is transferred to the material by thermal gradient-based convection, conduction, and radiation mechanisms from the soil surface, whereas the heating mechanisms of microwaves are dipolar polarization and conduction mechanisms.

Overall, on the basis of microwave heat principles [22], this depends on the dielectric properties of the soil, which exhibited a direct relationship with temperature variation. Mineralogical composition (Table 1) exhibited Al_2_O_3_ (34.2%) and SiO_2_ (32.3%) as the major phase, followed by Fe_2_O_3_ (15.1%) and other minerals (18.4%). Al and Fe oxides have dielectric properties up to approximately 30 times higher than those of SiO_2_ [22]. In addition, characteristics (e.g., mineral composition, soil texture, and moisture content) influence microwave penetration and molecular motion, thereby resulting in temperature difference in irradiated soil [27]. Therefore, the use of several dielectric materials makes a direct comparison of the results difficult.

### 3.2. Effect of Power Density in Microwaves on TPH Removal

Contaminated bulk soils were irradiated in a microwave for 15 and 20 min. Table 3 shows the TPH removal efficiency of the microwave treatment for different power densities. Contaminant removal efficiency from the soil after microwave treatment increased with time. Maximal TPH removal efficiency was observed with a microwave power density of 1.3 kW/kg at 20 min. When using a power density of 1.3 kW/kg, temperature was significantly increased to 310 °C for 20 min, and 80.2% of TPH were removed. Meanwhile, when using a low power density (0.5 kW/kg), removal efficiency was 45.2% at 20 min. The efficiency of TPH removal gradually increased with increasing microwave power and irradiation time because TPH consist of chemical substances with complex structures and large molecular weights [23]. This could be attributed to the generation of additional heat, which resulted in rapid molecular motion. On the other hand, weight loss (%) was observed in the microwave treatment, which was caused by TPH desorption in the soils. Weight loss could have also been caused by moisture and organic matter. This suggests that microwave power density influenced TPH removal. The results show that microwave power density and operating time could be significant in assessing changes in energy efficiency, and the main key factor in the remedial process.

### 3.3. Particle Size Effect

Soil particles were divided into two different particle sizes, the two contaminated soils types were measured as TPH fractions (C10–C40), and the results are shown in Table 4. TPH concentration in the soil increased with decreasing particle size. Hydrocarbon compounds were much more concentrated in the fine particle soil characterized by a higher specific surface area [28,29]. Falciglia et al. (2011) reported that an increase in concentration of approximately 6 times was observed for clay compared with that of coarse sand [21]. The presence of fine soil is a main factor for the hydrophobic adsorption of organic matter on the surface of fine soil particles. The data suggested that DRO (C10–C28) were more adsorbed in the soil, which was related to the presence of light hydrocarbons, while ORO (C28–C40) had relatively low concentrations. This indicates that contaminated soil influences the desorption of low molecular weight hydrocarbons. The fraction of light hydrocarbons can be transported to other environmental media because of its soluble and volatile compounds. Moreover, clay has inter-crystalline layers that can trap contaminants that penetrate the layers [30] and high porosity values (coarse soil, 34.6%; fine soil, 54.5%), which increase contaminant diffusion phenomena and represent limiting factors in desorption processes.

In order to study the influence of different particle sizes on the removal efficiency of TPH from artificially contaminated soil, various irradiation times were used. The residual concentration of contaminants and temperature as a function of irradiation time are shown in Table 5. Residual TPH concentration decreased with irradiation time, while temperature increased over time. For the coarse soil (>0.106 mm), removal efficiency reached a removal rate of 91.1% at 15 min, which was attributed to the rapid evaporation of contaminants from the soil particle surface. However, removal rate from fine soil (<0.106 mm) was low. When a time of 30 min was applied, a removal rate of 71.2% was found when lastly reaching the highest temperature of approximately 300 °C. Comparing the two different particle soil types, coarse soil had better removal temperatures below 300 °C, since TPH present in soil were formed by higher surface-to-mass ratios. Consequently, the lowest energy was required for coarse soil while the highest was required for fine soil. Such results showed that the influence of soil properties such as particle distribution in the remediation of contaminated soil is important.

**Figure 3:**
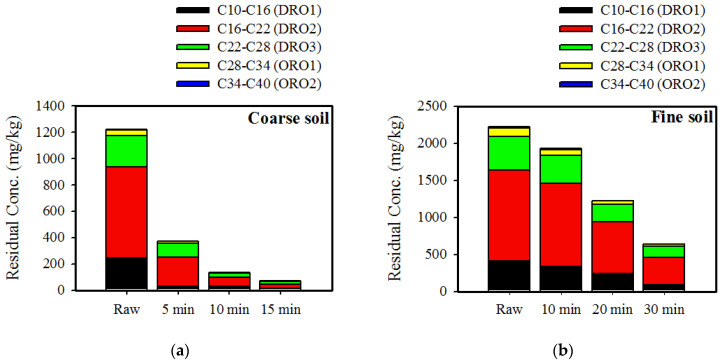
Residue concentration of different fractions in TPH as function of treatment time for different particle sizes. (**a**) Coarse soil (>0.106 mm) and (**b**) fine soil (<0.106 mm).

For the microwave treatment of coarse soil (Figure 3a), a significant decrease in residual TPH concentration in the soil in the fractions of C10–C16 (DRO1) and C16–C22 (DRO2) was observed at approximately 130 °C after 5 min. This depended on temperature increase and evaporation contaminant stripping. This indicated the desorption of the C10–C16 (DRO1) and C16–C22 (DRO2) fractions because of water vapor stripping. In addition, the C22–C28 (DRO3) and C28–C34 (ORO1) fractions slowly decreased when irradiation time was increased. Residual TPH concentrations in the fine soil had low removal efficiency and slowly decreased (Figure 3b). This indicated that different behaviors occurred. A high temperature could significantly remove TPH from the contaminated soil by the water distillation process using microwave treatment. However, fine soil could be influenced by evaporation contaminant stripping. Decreased vapor flows caused by low permeability were the reason for trapping contaminant to transfer to a gaseous phase, thereby limiting evaporation and decreasing removal efficiency. In addition, the internal temperature of the soil was higher than that of the soil surface, with the maximal difference observed approximately 66 °C (Table 5), which indicated that the inner layer of the soil was directly heated by microwaves and the heat transfer mechanism was heat conduction. Consequently, hot vapor flows were relatively concentrated by fine soil on the inner surface of the soil to form a superheated area. Increased temperature over time could modify the soil structure [24], thereby also increasing its permeability. This is because the generated heat was progressively transferred by conduction to the more distant layers.

### 3.4. Behavior of Different Fractions by Microwave Treatment

**Figure 4:**
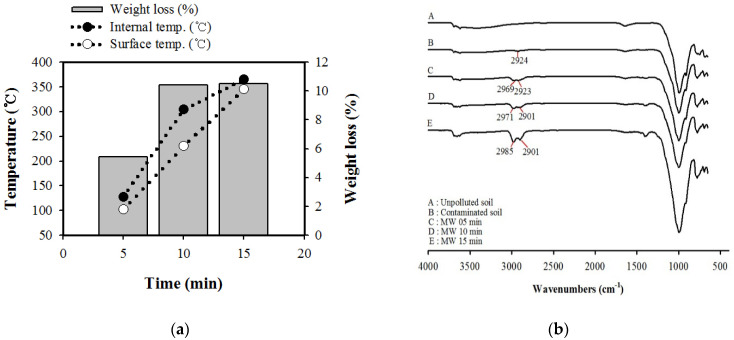
(**a**) Temperature, weight loss, and residual concentration from contaminated bulk soil with irradiation time. (**b**) FTIR spectra of soil sample before and after microwaves.

**Figure 5:**
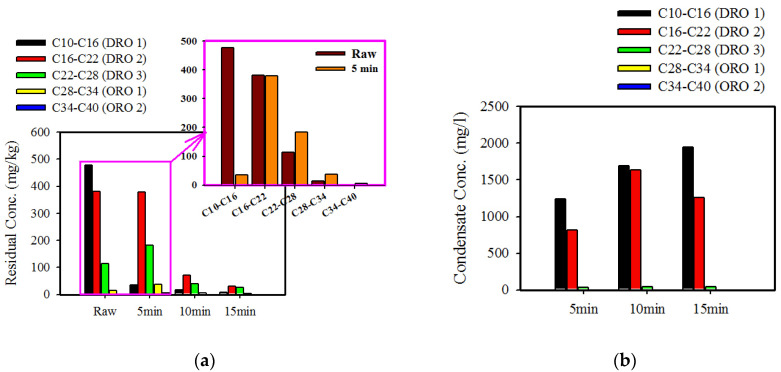
Concentration of different fraction on (**a**) soil and (**b**) condensate in TPH as a function of treatment time.

Bulk contaminated soil was investigated over time during microwave heating at 800 W. As expected, removal efficiency increased with time, and weight loss increased with temperature rising (Figure 4a). After 10 min of microwave treatment, weight loss and TPH removal efficiency were significantly increased at 300 °C. The surface chemistries of the contaminated and treated soil samples were studied using FTIR (Fourier-transform infrared spectroscopy). To verify the removal performance of different components during microwave treatment, the results of the functional groups of the contaminated soil detected by FTIR spectroscopy are shown in Figure 4b. Compared with uncontaminated soil, a new peak, at 2924 cm^−1^, arose in the FTIR curve of the contaminated soil, which was ascribed to the asymmetric C−H stretching vibrations of hydrocarbons [5,31]. The peak of artificially contaminated soil at 2924 cm^−1^ exhibited shifts to 2923 cm^−1^ at 5 min and disappeared in the irradiated soil, while bands at 2969, 2971, and 2985 cm^−1^ exhibited shifts with increased irradiation time, thereby suggesting that the C–H in the saturated C stretching vibration and alkyl groups shifted. The FTIR results indicate the fundamental vibrations and associated of organic compounds formed as contaminated soil of incomplete combustion. After microwave treatment, these peaks were still present but became weaker. The surface functional groups of contaminated soil influenced by microwave irradiation and changes in the hydrocarbon fraction would have an impact on contaminant removal. According to the microwave treatment results, desorption and adsorption behavior occurred simultaneously, as shown in Figure 5. The behavior of different TPH fractions showed that the desorption process might have been affected by van der Waals and hydrophobic forces [31]. Because a decrease in lightest TPH fraction DRO1 (C10–C16) occurred with an increase in treatment time, whereas DRO3 (C22–C28) and ORO1 (C28–C34) fractions increased, the concentration decreased.

Residual TPH concentrations of each fraction are shown in Figure 5 in order to observe the desorption behavior of five carbon number-based fractions. The contaminants adsorbed onto the soil were removed by evaporation because the water solubility of the hydrocarbons increased, and the saturated vapor pressure of the hydrocarbons increased with increasing temperature, thereby contributing to an effective transition of the contaminant to the gaseous phase. As shown in Figure 5a, a decrease in the lightest TPH fraction (C10–C16) occurred as treatment time increased, whereas the C22–C28 (114.4 to 183.0 mg/kg) and C28–C34 (14.1 mg/kg to 38.3 mg/kg) fractions increased (Table A1). The evaporation of the C10–C16 fraction produced solubilization and re-adsorption, while the other portion was released in the form of gas. The increase in temperature could destroy the force existing between soil and contaminants. The condensate concentration of each fraction is shown in Figure 5b. The microwave treatment caused major desorption of the light DRO fractions, and an increase in the concentrations of the C10–C16 and C16–C22 fractions was recorded with increasing remediation time.

## 4. Discussion

TPH are key evaluation indicators for establishing target cleanup levels for petroleum contaminated soil. The United States Environmental Protection Agency [32] and the Total Petroleum Hydrocarbon Criteria Working Group (TPHCWG) [33] developed fraction-based approaches as an assessment of human health risks. Cho et al. (2019) showed that TPH can degrade and partition during chemical weathering; gasoline-range organics (C6–C10) and diesel-range organics (DRO; C10–C28), which are the more soluble and volatile compounds, can be transported to other environmental media, while oil-range organics (ORO; C28–C40), which are relatively non-mobile and recalcitrant, remain near the point of release [34]. A chemical understanding of TPH is helpful in designing an optimal remediation process and for estimating the environmental impact of these TPH [35]. TPH immobility in soils makes it challenging to dispose of contaminated soils.

To develop effective and rapid remediation for TPH disintegration, extensive research on developing an efficient remediation process for soils contaminated with TPH was conducted [36]. In particularly, with increasing awareness of post-remediation soil qualities, lower temperatures may be preferred over burn off at high temperatures, because high temperatures could adversely affect soils by removing soil components during thermal treatment. Thus, microwave heating can be used as an alternative to minimize the detrimental effects of thermal treatment. The influence of soil texture and moisture has largely been accessed on TPH removal efficiency, and the behavior of five carbon number-based TPH fractions has not been extensively studied. Since there is a growing interest in minimizing the negative effects of thermal treatment on soil qualities, changes in soil properties and the behavior of different fractions of TPH in soils after microwave heating need to be studied.

In this study, TPH desorption behavior from artificially contaminated soil was studied with soil properties such as particle size in order to remediate diesel fuel contaminated soils. The microwave power density for TPH removal was affected by soil particle distribution in the contaminated soil. In addition, soil dielectric properties and moisture could affect the optimal microwave power density. Thus, in order to apply microwave heating treatment to field remediation, the treatment operating conditions should be determined after considering soil properties such as mineral composition and, soil texture, and microwave power density used for operation. Therefore, the scale-up application of microwave treatments for a full-scale can offer effective TPH remediation activities.

## 5. Conclusions

The microwave desorption treatment of TPH-contaminated soil was investigated with regard to the desorption behavior of five carbon number-based TPH fractions. In particular, this study observed microwave heating behavior between two different particle size soil and bulk soil. Operating microwave power density influenced the final temperature reachable during heating. For low operating power density application, microwave effectiveness is limited due to the soil dielectric properties, which exhibited a direct relationship with temperature variation, since microwave heating mechanisms are dipolar polarization. This suggests that microwave power density and operating time could be significant in assessing changes in energy efficiency and the main key factor in the remedial process.

Soil particle distribution could be attributed to permeability, which significantly influenced the evaporation of the soil sample during microwave treatment. Comparing the two different particle soil types, coarse soil has better removal temperatures below 300 °C, since TPH present in soil were formed by higher surface-to-mass ratios. The results indicate that the lowest energy was required for coarse soil while the highest was required for fine soil. In addition, decreased vapor flows caused by low permeability were the reason for trapping contaminants to transfer to the gaseous phase, thereby limiting evaporation and decreasing removal efficiency. Such results show that the influence of soil properties such as particle distribution in the remediation of contaminated soil is important.

Residual TPH concentration decreased when irradiation time was increased with a removal rate that depended on soil temperature variation. The stripping dominated the removal of hydrocarbons at 100~200 °C, where light hydrocarbons were removed with soil moisture, of which evaporation was the solvent, and the mass transfer of hydrocarbons to the gas phase was also enhanced by the distillation process. The surface functional groups of contaminated soil were influenced by microwave irradiation, and changes in the hydrocarbon fraction had an impact on contaminant removal.

## Figures and Tables

**Table 1:** Chemical compositions (wt.%) of soil sample.

Component	Al_2_O_3_	SiO_2_	Fe_2_O_3_	K_2_O	CaO	MgO	Na_2_O
Content (%)	34.2	32.3	15.1	11.7	1.73	1.56	1.25

**Table 2:** Soil sample properties and characteristics.

Parameter	pH	Porosity (%)	Specific Surface Area (m^2^/g)	Organic Matter (g/kg)	Moisture Content (%)
Value	6.9	26.9	1.52	1.63	4.57

**Table 3:** Total petroleum hydrocarbon (TPH) removal (%), temperature (°C) and weight loss (%) from contaminated soil with irradiation time (15 and 20 min, respectively).

kW/kg	15 min			20 min		
	Removal (%)	Temp. (°C)	Weight Loss (%)	Removal (%)	Temp. (°C)	Weight Loss (%)
1.3	74.4	240.7	10.5	80.2	310.8	12.2
0.9	60.6	196.3	10.4	72.2	256.6	11.7
0.5	15.8	136.4	9.88	45.2	189.6	10.3

**Table 4:** Distribution of TPH on different particle soil types as TPH fractions after contamination procedure.

Soil Type	TPH Fraction (mg/kg)					Total (mg/kg)
	C10–C16	C16–C22	C22–C28	C28–C34	C34–C40	
Coarse soil (>0.106 mm)	245.1	693.1	237.6	42.3	4.02	1222.1
	20.1%	56.7%	19.4%	3.46%	0.33%	100%
Fine soil (<0.106 mm)	414.5	1222.5	456.8	107.6	21.6	2223.0
	18.6%	55.0%	20.6%	4.84%	0.97%	100%

**Table 5:** Removal efficiency and temperature as a function of time for different particle sizes.

**Soil Type**	**Time (min)**	**Temperature (°C)**		**Removal Efficiency (%)**	**Residual Concentration (mg/kg)**
		**Internal**	**Surface**		
Coarse soil (>0.106 mm)	5.0	132.0	125.8	59.9	396.1
	10.0	195.0	191.5	88.5	113.7
	15.0	255.0	231.0	91.1	88.1
**Soil Type**	**Time (min)**	**Temperature (°C)**		**Removal Efficiency (%)**	**Residual Concentration (mg/kg)**
		**Internal**	**Surface**		
Fine soil (<0.106 mm)	10.0	150.0	128.0	13.1	1931.9
	20.0	280.0	232.0	45.0	1222.1
	30.0	370.0	304.1	71.2	639.9

## Appendix A

**Table A1:** Removal efficiency and temperature as a function of time for the different particle size.

**Soil Type**	**Time (min)**	**Temperature (°C)**		**Removal Efficiency (%)**	**Residual Concentration (mg/kg)**
		**Internal**	**Surface**		
+0.106 mm	5.0	132	125.8	59.9	396.1
	10.0	195	191.5	88.5	113.7
	15.0	255	231	91.1	88.1
**Soil Type**	**Time (min)**	**Temperature (°C)**		**Removal Efficiency (%)**	**Residual Concentration (mg/kg)**
		**Internal**	**Surface**		
−0.106 mm	10.0	150	128	13.1	1931.9
	20.0	280	232	45.0	1222.1
	30.0	370	304.1	71.2	639.9

## References

[1] Karer J., Wawra A., Zehetner F., Dunst G., Wagner M., Pavel P.-B., Puschenreiter M., Friesl-Hanl W., Soja G. (2015). Effects of Biochars and Compost Mixtures and Inorganic Additives on Immobilisation of Heavy Metals in Contaminated Soils. Water Air Soil Pollut..

[2] Das A.J., Kumar R. (2016). Bioremediation of petroleum contaminated soil to combat toxicity on Withania somnifera through seed priming with biosurfactant produci ng plant growth promoting rhizobacteria. J. Environ. Manag..

[3] Pinedo J., Ibañez R., Lijzen J., Irabien A. (2013). Assessment of soil pollution based on total petroleum hydrocarbons and individual oil substances. J. Environ. Manag..

[4] Falciglia P.P., De Guidi G., Catalfo A., Vagliasindi F.G.A. (2016). Remediation of soils contaminated with PAHs and nitro-PAHs using microwave irradiation. Chem. Eng. J..

[5] Li D.-C., Xu W.-F., Mu Y., Yu H.-Q., Jiang H., Crittenden J. (2018). Remediation of Petroleum-Contaminated Soil and Simultaneous Recovery of Oil by Fast Pyrolysis. Environ. Sci. Technol..

[6] Huesemann M.H., Truex M.J. (1996). The role of oxygen diffusion in passive bioremediation of petroleum contaminated soils. J. Hazard. Mater..

[7] Rothermich M.M., Hayes L.A., Lovley D. (2002). Anaerobic, Sulfate-Dependent Degradation of Polycyclic Aromatic Hydrocarbons in Petroleum-Contaminated Harbor Sediment. Environ. Sci. Technol..

[8] Cho K., Myung E., Kim H., Park C., Choi N., Park C. (2020). Effect of Soil Washing Solutions on Simultaneous Removal of Heavy Metals and Arsenic from Contaminated Soil. Int. J. Environ. Res. Public Health.

[9] Ouriache H., Arrar J., Namane A., Bentahar F. (2019). Treatment of petroleum hydrocarbons contaminated soil by Fenton like oxidation. Chemosphere.

[10] Da Rocha U.N., Van Elsas J.D., Van Overbeek L.S. (2010). Real-time PCR detection of Holophagae (Acidobacteria) and Verrucomicrobia subdivision 1 groups in bulk and leek (Allium porrum) rhizosphere soils. J. Microbiol. Methods.

[11] Kang C.-U., Kim D.-H., Khan M.A., Kumar R., Ji S.-E., Choi K.-W., Paeng K.-J., Park S., Jeon B.-H. (2020). Pyrolytic remediation of crude oil-contaminated soil. Sci. Total. Environ..

[12] Samaksaman U., Kuo J.-H., Peng T.-H., Wey M.-Y. (2015). Determination of Emission Characteristics during Thermal Treatment of Lube Oil and Heavy Metal Co-Contaminated Soil by Fluidized Bed Combustion. J. Environ. Eng..

[13] Júnior B.R.D.C.L., Tribst A.A.L., Grant N.J., Yada R.Y., Cristianini M. (2017). Biophysical evaluation of milk-clotting enzymes processed by high pressure. Food Res. Int..

[14] Zhou H., Hu L., Wan J., Yang R., Yu X., Li H., Chen J., Wang L., Lu X. (2016). Microwave-enhanced catalytic degradation of p-nitrophenol in soil using MgFe2O4. Chem. Eng. J..

[15] Cho K., Kim H., Myung E., Purev O., Choi N., Park C. (2020). Recovery of Gold from the Refractory Gold Concentrate Using Microwave Assisted Leaching. Metals.

[16] Zhou D., Randall C.A., Pang L.-X., Wang H., Guo J., Zhang G.-Q., Wu X.-G., Shui L., Yao X. (2011). Microwave Dielectric Properties of Li2WO4 Ceramic with Ultra-Low Sintering Temperature. J. Am. Ceram. Soc..

[17] Robinson J., Kingman S.W., Snape C., Bradshaw S., Bradley M., Shang H., Barranco R. (2010). Scale-up and design of a continuous microwave treatment system for the processing of oil-contaminated drill cuttings. Chem. Eng. Res. Des..

[18] Atienza-Martínez M., Ábrego J., Gea G., Marías F. (2020). Pyrolysis of dairy cattle manure: Evolution of char characteristics. J. Anal. Appl. Pyrolysis.

[19] Falciglia P.P., Scandura P., Vagliasindi F.G.A. (2017). Modelling and preliminary technical, energy and economic considerations for full-scale in situ remediation of low-dielectric hydrocarbon-polluted soils by microwave heating (MWH) technique. J. Soils Sediments.

[20] Sivagami K., Padmanabhan K., Joy A.C., Nambi I.M. (2019). Microwave (MW) remediation of hydrocarbon contaminated soil using spent graphite – An approach for waste as a resource. J. Environ. Manag..

[21] Falciglia P., Giustra M., Vagliasindi F.G.A. (2011). Low-temperature thermal desorption of diesel polluted soil: Influence of temperature and soil texture on contaminant removal kinetics. J. Hazard. Mater..

[22] Falciglia P., Vagliasindi F.G.A. (2015). Remediation of hydrocarbon polluted soils using 2.45GHz frequency-heating: Influence of operating power and soil texture on soil temperature profiles and contaminant removal kinetics. J. Geochem. Explor..

[23] Buttress A., Binner E.R., Yi C., Palade P., Robinson J., Kingman S.W. (2016). Development and evaluation of a continuous microwave processing system for hydrocarbon removal from solids. Chem. Eng. J..

[24] Malafronte L., Lamberti G., Barba A.A., Raaholt B., Holtz E., Ahrné L. (2012). Combined convective and microwave assisted drying: Experiments and modeling. J. Food Eng..

[25] Falciglia P.P., Urso G., Vagliasindi F.G.A. (2013). Microwave heating remediation of soils contaminated with diesel fuel. J. Soils Sediments.

[26] E Clark D., Folz D.C., West J.K. (2000). Processing materials with microwave energy. Mater. Sci. Eng. A.

[27] Sengwa R., Soni A. (2008). Dielectric properties of some minerals of western Rajasthan. Indian J. Radio Space Phys..

[28] Amellal N., Portal J.-M., Berthelin J. (2001). Effect of soil structure on the bioavailability of polycyclic aromatic hydrocarbons within aggregates of a contaminated soil. Appl. Geochem..

[29] Martín F.J.S., Fernandez-Salguero P.M., Merino J.M. (2011). Aryl hydrocarbon receptor-dependent induction of apoptosis by 2,3,7,8-tetrachlorodibenzo-p-dioxin in cerebellar granule cells from mouse. J. Neurochem..

[30] LaGrega M.D., Buckingham P.L., Evans J.C. (2010). Hazardous Waste Management.

[31] Li F., Zhang Y., Wang S., Li G., Yue X., Zhong D., Chen C., Shen K. (2020). Insight into ex-situ thermal desorption of soils contaminated with petroleum via carbon number-based fraction approach. Chem. Eng. J..

[32] Boon K.A., Ramsey M.H. (2012). Judging the fitness of on-site measurements by their uncertainty, including the contribution from sampling. Sci. Total Environ..

[33] Park I.-S., Park J.-W. (2010). A novel total petroleum hydrocarbon fractionation strategy for human health risk assessment for petroleum hydrocarbon-contaminated site management. J. Hazard. Mater..

[34] Cho E., Park M., Hur M., Kang G., Kim Y., Kim S. (2019). Molecular-level investigation of soils contaminated by oil spilled during the Gulf War. J. Hazard. Mater..

[35] Liu Z., Zhang S., Hu D., Zhang Y., Lv H., Liu C., Chen Y., Sun J. (2019). Paraffin/red mud phase change energy storage composite incorporated gypsum-based and cement-based materials: Microstructures, thermal and mechanical properties. J. Hazard. Mater..

[36] Falciglia P.P., Vagliasindi F.G.A. (2015). Techno-economic analysis of hydrocarbon-polluted soil treatment by using ex situ microwave heating: Influence of soil texture and soil moisture on electric field penetration, operating conditions and energy costs. J. Soils Sediments.

